# Downregulation of REST in the cochlea contributes to age-related hearing loss via the p53 apoptosis pathway

**DOI:** 10.1038/s41419-022-04774-0

**Published:** 2022-04-13

**Authors:** Hongchen Li, Mingshun Lu, Haiwei Zhang, Shengnan Wang, Fei Wang, Xueya Ma, Jiaxi Liu, Xinyu Li, Haichao Yang, Haitao Shen, Ping Lv

**Affiliations:** 1 Department of Pharmacology, Hebei Medical University, Shijiazhuang, Hebei 050017 China; 2 Center for Innovative Drug Research and Evaluation, Institute of Medical Science and Health, Hebei Medical University, Shijiazhuang, Hebei 050017 China; 3 The Key Laboratory of Neural and Vascular Biology, Ministry of Education, Hebei Medical University, Shijiazhuang, Hebei 050017 China; 4 Lab of Pathology, Hebei Medical University, Shijiazhuang, Hebei 050017 China

**Keywords:** Cochlea, Apoptosis

## Abstract

Age-related hearing loss (AHL) is the most common sensory disorder amongst the elderly population. Although the degeneration of spiral ganglion neurons (SGNs) and hair cells (HCs) is considered to play a critical role in AHL, the mechanism has not been fully outlined. The repressor element 1-silencing transcription factor (REST) has recently been associated with mediating cell death in neurodegenerative diseases. However, whether REST induces degeneration of cochlear HCs and SGNs to contribute to AHL remains unknown. Here, we report that REST expression was decreased in HCs and SGNs in AHL mice. Conditional deletion of *Rest* in HCs and SGNs of 2-month-old mice resulted in hearing loss accompanied by the upregulation of p53, TNFR1(tumor necrosis factor receptor-1), and cleaved caspase-3. The p53 inhibitor pifithrin-α significantly attenuated SGN and HC damage and rescued hearing impairment in *Rest* cKO mice. Furthermore, downregulation of REST by H_2_O_2_ treatment induced apoptosis in the House Ear Institute Organ of Corti 1 cell, through the upregulation of p53. In contrast, overexpression of REST reversed the changes in p53 expression. In addition, REST was further shown to bind directly to the p53 promoter site, thereby inhibiting the effect of p53. Finally, in aged mice, the p53 inhibitor significantly reduced loss of HCs and SGNs, and subsequently improved hearing. In summary, our findings indicate that REST has a protective role in AHL, and that its deficiency upregulates p53 and induces cochlear cell apoptosis, which that leads to deafness.

## Introduction

Age-related hearing loss (AHL), also known as presbycusis, is the most prevalent sensory deficit in the elderly population [1]. Characterized by a decrease in high-frequency hearing sensitivity, AHL refers to a symmetrical, progressive, age-dependent decline in binaural auditory function. Hearing disability may impair auditory sensitivity and speech perception in older adults, leading to a reduced quality of life and increased incidence of psychiatric disorders, such as social isolation and depression [2]. Recent studies have identified AHL as a high-risk factor for cognitive decline and dementias, including Alzheimer’s disease [3–5]. Degeneration of cochlear spiral ganglion neurons (SGNs) and hair cells (HCs) has been shown to play a vital role in AHL [6–9], although the underling molecular mechanism has not yet been fully clarified.

HCs and SGNs are vital for transmitting auditory signals. HCs are located in the cochlea and convert sound-evoked mechanical signals into electrical signals for transmission to the SGNs. These transmit the electrical signals to the brain in a manner that preserves the amplitude, frequency, and temporal characteristics of the sound information. Due to the fact that HCs and SGNs do not regenerate in the mature mammalian cochlea, their degeneration leads to irreversible hearing loss.

Repressor element 1-silencing transcription factor (REST) is an essential transcriptional regulator. It contains a DNA binding domain, which can bind to a specific 21 base pair (bp) consensus sequence (RE-1) to repress the transcription of target genes [10–12]. REST was originally found to be involved in neurogenesis, differentiation, axonal growth, and vesicular release [13, 14]. Deletion of the *Rest* gene results in the genomic instability of neural progenitor cells and neurons, which may lead to the premature differentiation of neural progenitor cells [15]. Recently, emerging evidence has suggested that REST expression and activity are decreased in Alzheimer’s disease [16] and Parkinson’s disease [17], and that neuron apoptosis caused by REST downregulation contributes to these pathological process. Moreover, REST upregulation has been recorded in the human brain during physiological aging [18]. In vitro, REST directly facilitates cellular senescence through interaction with autophagic machinery. Specific inhibition of REST impairs autophagy and mitochondrial function [19]. These studies suggest that REST is important in both physiological aging and age-related diseases. However, it remains unknown whether REST-mediated degeneration of SGNs and HCs is implicated in AHL.

This study aimed to describe the role of REST in AHL. We created a mouse model with a conditional REST knockout (cKO) in the cochlea. The results show that the expression of REST was decreased in SGNs and HCs in AHL mice. Deletion of REST induced p53-dependent apoptosis in SGNs and HCs, resulting in hearing loss in *Rest* cKO mice. Overexpression of REST alleviated apoptosis by downregulating p53 in vitro. Administration of a p53 inhibitor reduced damage to SGNs and HCs, and rescued hearing impairment in *Rest* cKO mice and AHL mice.

## Materials and methods

### Animal

All experimental animal protocols were performed following the Animal Care and Ethical Committee of Hebei Medical University (Shijiazhuang, China). C57BL/6 mice (C57) were obtained from the Charles River Labs. *Atoh1-Cre: Rest*^*flox/flox*^ conditional knockout mice (*Rest* cKO) were created by crossing *Atoh1-Cre* (Jackson Laboratories, Bar Harbor, ME, stock No. 011104) with *Rest*^*flox/flox*^ (kindly provided by Prof. Buckley and Prof. Gamper) mice. *Rest* cKO mice were in C57 background. BALB/c mice were purchased from Beijing Vital River Laboratory Animal Technology Co. Ltd (Beijing, China). PASS Sample Size Software was used to evaluate sample size. The experimental mice were grouped by a simple random sampling method and the relative assays were performed blindly. The mice were bred in-house under a 12:12 h light-dark cycle.

### Auditory brainstem response (ABR) testing and Distortion product otoacoustic emissions (DPOAE)

ABR measurements were performed as previously described [20]. The mice were anesthetized with ketamine (100 mg/kg) and xylazine (10 mg/kg). Platinum needle electrodes were inserted subcutaneously at the vertex of the head [6], ipsilateral mastoid (recording), and contralateral rear leg of the mouse (ground). At 8, 12, 16, 20, 24, 28, and 32 kHz, tone stimuli were emitted from a 20 dB, with an attenuation interval of 5 dB. ABRs were measured using a System III workstation (Tucker Davis Technologies, Alachua, FL, USA) in an IAC BioSigRP Soundbooth (GM Instruments, Irvine, UK). The hearing threshold was defined as the lowest sound intensity required to generate a reproducible ABR waveform.

To measure DPOAE, two pure tones, f1 and f2 were generated through a Tucker-Davis Technologies (TDT) RZ6 system under the control of TDT BioSigRZ software driving electrostatic speakers via separate channels. Frequencies were collected at 8, 12, 16 28, and 32 kHz to measure the response signals to the 2f1-f2, with a ratio of 1.2 for f2/f1. Hearing thresholds were defined as the averaged signal for each identified frequency tested and compared to the corresponding frequency of the controls.

### Morphometry and counting of SGNs and HCs

The mice were sacrificed, and the cochleae were fixed in 4% paraformaldehyde (PFA) overnight at 4 °C, and then decalcified in 10% EDTA (#798681, Sigma, Darmstadt, Germany) in PBS for 2–3 days at 4 °C. The decalcified cochleae were processed using a sucrose gradient and embedded in an optical cutting temperature (OCT) compound (Tissue-Tek) for cryosectioning. Cochlear sections were stained with hematoxylin and eosin to evaluate SGN morphometry and density. SGN numbers were counted in the apex, middle, and base of the cochlea from each section.

HC preparations were performed for immunofluorescence staining. Cells displaying Myo7A labelling were quantified as the number of positive cells per 100 μm of basilar membrane length, from the apex, middle, and base for HC-counts.

### Immunofluorescence

Cochlear sections were prepared and stained following a previously established protocol [20]. The specimens were incubated with related primary antibodies overnight at 4°C. Mouse anti-cleaved caspase-3 antibody (#G7481, Promega, Madison, WI, USA; 1:100), rabbit anti-REST antibody (#ab21635, Abcam, Cambridge, UK, 1:200), mouse anti-Tuj1 (#801202, Biolegend, CA, USA; 1:100) or rabbit anti-Myo7A antibody (#25-6790, Proteus BioSciences, CA, USA; 1:50) were used in this experiment. The sections were rinsed with phosphate-buffered saline (PBS) for 3 times then incubated with Alexa 488-conjugated or Alexa 568-conjugated secondary antibodies (#115-545-003; 115-165-003; 111-545-003; 111-165-003, Jackson Immuno Research, PA, USA; 1:200) for 2 h at room temperature (22 °C–24 °C). The sections were rinsed with PBS and stained with 4,6-diamidino-2-phenylindole dihydrochloride (DAPI, #D9542, Sigma, Darmstadt, Germany) solution for 10 min at room temperature, and mounted using antifade mounting medium (# P36934, Invitrogen, CA, USA). Images were captured with a Leica TCS SP5 confocal microscope (Leica Microsystems, Wetzlar and Mannheim, Germany), analyzed with Leica LAS AF Lite, and processed with ImageJ and Photoshop CS5 (Adobe, San Jose, CA).

### Quantitative real-time PCR

The total RNA sample from mouse cochlea (4-5 mice were used for each sample) was extracted according to a previously established method [21] using the RNAeasy Micro Kit (Qiagen, Hilden, Germany). As in the reverse-transcription kit manual (Takara Bio Inc., Dalian, China), RNA were synthesized to cDNA using the random hexamers and superscript II reverse transcriptase. Quantitative PCR was carried out by using a TB GREEN Kit (Takara Bio Inc., Dalian, China) with a two-step cycling program using a Bio-Rad CFX Connect Real Time PCR system. Primer sequences are listed in Table 1. The relative gene expression was analyzed by calculating the respective *GAPDH* using the formula: 2^−ΔΔCt^.

**Table 1:** The primers sequences for RT-PCR.

GAPDH forward	5ʹ-TTGATGGCAACAATCTCCAC-3ʹ
GAPDH reverse	5ʹ-CGTCCCGTAGACAAAATGGT-3ʹ
REST forward	5ʹ-TCTCCAAGGCTGAGTTTTCAGT-3ʹ
REST reverse	5ʹ-ACATTAACTCCCGAGGATTTGC-3ʹ
BID forward	5ʹ-CCAAAGCCCTTGATGAGGTG-3ʹ
BID reverse	5ʹ-GCAAAGATGGTGCGTGACTG-3ʹ
BAX forward	5ʹ-TGCTGATGGCAACTTCAACTG-3ʹ
BAX reverse	5ʹ-AAGTCCAGTGTCCAGCCCAT-3ʹ
FADD forward	5ʹ-TGGGGGAAGACACCATCTCA-3ʹ
FADD reverse	5ʹ-CTACCCTTTAAACCACAGTCCTCAC-3ʹ
DAXX forward	5ʹ-AAGCCTCCTCGGAATCTGGT-3ʹ
DAXX reverse	5ʹ-CGTCATCATCATCGTCATCATC-3ʹ
FAS forward	5ʹ-ACAAGTTCTGTGCCACCATTG-3ʹ
FAS reverse	5ʹ-TTGTCCATGTACTCCTTCCCTTC-3ʹ
P53 forward	5ʹ-GGCGTAAACGCTTCGAGATG-3ʹ
P53 reverse	5ʹ-CTTCAGGTAGCTGGAGTGAGC-3ʹ
TNFR1 forward	5ʹ-GCTGTTGCCCCTGGTTATCT-3ʹ
TNFR1 reverse	5ʹ-ATGGAGTAGACTTCGGGCCT-3ʹ

### Western blot

Cochlear tissues (4-5 mice were used for each sample) or cells were harvested and homogenized in a 50 μL lysis buffer solution (50 mM Tris-HCl pH 7.5, 150 mM NaCl, 2 mM EDTA, 1% SDS, 1% TritonX-100). The proteins were fractionated by 12% Sodiumdodecyl sulfate-polyacrylamide gel electrophoresis (SDS-PAGE) and transferred to a polyvinylidene difluoride membrane (Millipore, MA, USA). Membranes were blocked with Tris buffered saline with tween 20 (TBST) containing 5% dry milk for 2 h at room temperature and probed with primary antibodies overnight on a rocker at 4 °C. Fluorescent secondary antibody (IRDye800CW, LI-COR, Biosciences, NE, USA) was then applied for 90 min, at room temperature. The immunoreactive bands were quantified with ImageLab 4.0 (Bio-Rad, CA, USA). All the western blotting experiments were examined independently at least three times, with the same trends seen each time.

### Plasmids and siRNA transfection

The HEI-OC1(House Ear Institute Organ of Corti 1) mouse inner ear cell line (Biofeng Lab) were maintained in Dulbecco’s Modified Eagle Medium (DMEM) with 10% fetal bovine serum (Gibico, Sydney, Australia), supplemented with antibiotics at 33 °C, with 10% CO_2_. The full-length *Rest* gene (NM_011263.2) was synthesized from Sangon Biotech (Shanghai, China) and cloned to vector pcDNA3.1 to generate plasmid pcDNA3.1-REST by standard molecular biology techniques; this was confirmed by sequencing containing NcoI and XbalI restrictive endonuclease sites. The expression of REST was silenced by using two oligonucleotides of small interfering RNAs targeting REST (siRESTa: CCGGTTGGTATTGTAGCCA; siRESTb: TTGGCGCTGTATATTTCTG). The cultured HEI-OC1 cells were washed and transfected with siRNAs or plasmids using Lipofectamine^TM^ 2000 (Thermo Fisher, Waltham, MA, USA) in Opti-MEM, as per the manufacturer’s instructions.

### Cell viability assay

The viability of HEI-OC1 cells was assayed with a cell counting kit (CCK-8, Sangon Biotech, Shanghai, China). Cells were cultured in 96-well plates at a density of 1 × 10^4^ per well and treated with various concentrations of H_2_O_2_ (100, 200, 300, 400, 600, and 800 μM) for 1 h, then 10 μL CCK8 solution was added, the solution was incubated at 37 °C for 4 h. The optical density of each well was measured at 450 nm using a FLUO star Omega microplate reader (BMG Labtech GmbH, OFFENBURG, Germany).

### Chromatin immunoprecipitation (ChIP)

ChIP assay was performed based on a previous study [16]. Briefly, HEI-OC1 cells were washed with PBS and cross-linked using 1% formaldehyde (Sigma, USA) for 10 min, at room temperature. Genomic DNA of cell lysate was sonicated to produce nucleotide fraction of 200–1000 base pairs and then centrifuged at 13,000 rpm at 4°C. The sediments were dissolved with ChIP dilution buffer (ChIP Assay Kit, Beyotime Biotechnology, Beijing, China) and mixed with Protein A/G Sepharose beads (Invitrogen, USA), followed by centrifugation at 1000 rpm at 4 °C. The supernatant was incubated with anti-REST or anti-IgG primary antibody overnight [#22-579, Millipore, USA] at 4°C. The antibody-conjugated beads were washed, the elute was incubated for 4 h, at 65 °C, to dissociate the conjugated DNA. DNA was isolated using a DNA purification kit (#B518141, Sangon Biotech, Shanghai, China), followed by ChIP-PCR. The nucleotide sequences of primers used for amplification of immunoprecipitated DNA were p53: forward 5ʹ- CTTCAGAAAACAGAGGAACAGACTG-3ʹ, reverse 5ʹ-TCCTGAGT AACTGGCATTACAGGT-3ʹ; SNAP25: forward 5ʹ-AGACTCCTT TGCAGACAATTTCCT-3ʹ, reverse 5ʹ-CAACACAGAAGATTTCCAC AAGTAGAC-3ʹ. Data were collected from three independent experiments.

### Assessment of cell apoptosis

The cell apoptosis was measured using an Annexin V-FITC/7-AAD (#559763, BD Biosciences, NJ, USA) staining kit. HEI-OC1 cells were transfected with a REST plasmid or control vector, and then treated with H_2_O_2_. After treatment, cells were resuspended in binding buffer and stained with FITC-labeled Annexin V and 7-AAD, for 20 min at room temperature, in the dark. Cell apoptosis was detected using FACS Calibur (BD Biosciences, NJ, USA) and data were analyzed by using the FlowJo software v7.6.1 (FlowJo, LLC, NJ, USA).

Terminal Deoxynucleotidyl Transferase (TdT)-Mediated dUTP Nick-End Labeling (TUNEL) assay was used to detect the apoptotic cells, according to the manufacturer’s instructions. Cochlear sections were permeabilized in 0.3% Triton X-100 for 60 min, and then blocked with a fluorometric terminal deoxytransferase solution for 1 h, at 37 °C. The sections were then incubated with anti-Tuj1 primary antibody overnight at 4 °C. They were then stained with a Cy3-conjugated secondary antibody for 2 h, at room temperature. Finally, the nuclei were stained with DAPI, and fluorescent images were taken using a Leica TCS SP5 confocal fluorescent microscope.

### In vivo drug administration

For pifithrin-α (PFT-α) administration in *Rest* cKO mice: pifithrin-α, a p53 inhibitor, was obtained from MCE (#63208-82-2, NJ, USA). PFT-α was dissolved in DMSO, diluted with 0.9% saline, and administered intraperitoneally to 2-month-old *Rest* cKO mice. The injections were given at a dosage of 2.2 mg/kg every two days, for 30 days. ABR was recorded before and after treatment with the drug to assess hearing function. The cochleae were also removed after the treatment to evaluate morphological changes of the SGNs.

For pifithrin-α administration in AHL mice: the 8-month-old C57 mice received either saline or pifithrin-α, at a dosage of 1.1 mg/kg, 2.2 mg/kg, intraperitoneally every other day, for 30 days. ABR was measured before and after treatment with pifithrin-α to assess hearing function. HCs and SGNs were isolated for evaluating morphological changes and apoptosis.

### Statistical analysis

All data are presented as the mean ± SEM. Statistical analyses were performed using GraphPad Prism (version 8.0.2.; GraphPad Software, Inc., San Diego, CA, USA). Student’s t-test was performed for comparisons between the two groups. For comparisons more than two groups, statistical analysis was performed using one-way analysis of variance [22] followed by Bonferroni post hoc test. The Kruskal–Wallis test was performed for the comparisons of data with non-normal distribution or heterogeneity of variance. *, **, and *** indicated statistically significant results compared to the appropriate controls and indicated *P* < 0.05, *P* < 0.01, and *P* < 0.001, respectively.

## Results

### Degeneration of SGNs and HCs in AHL mice

**Fig. 1:**
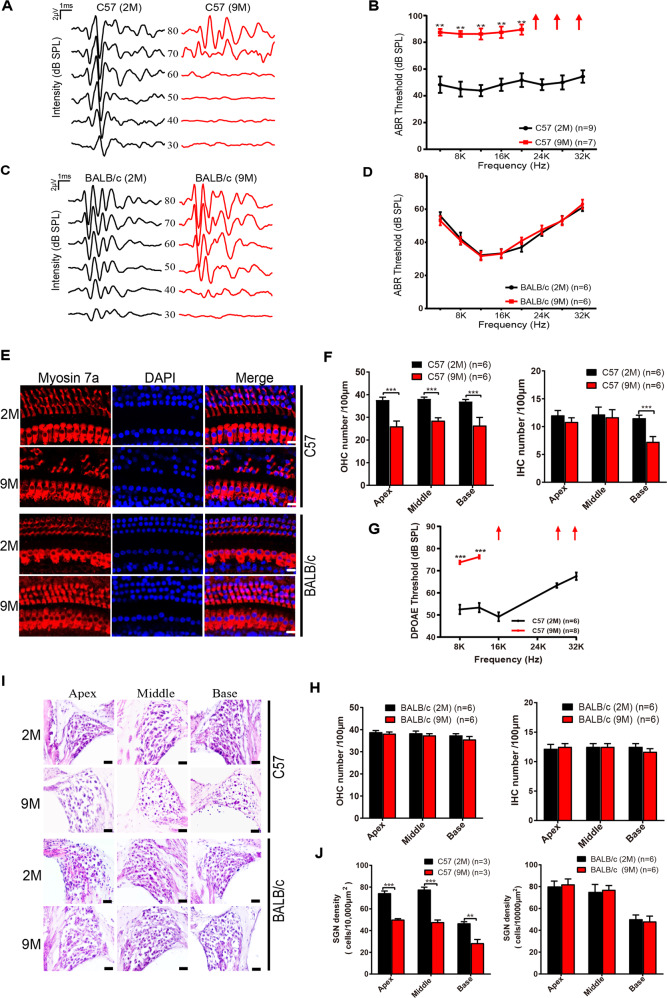
Degeneration of SGNs and HCs in AHL mice. **A** Representative ABR waveforms, in response to clicking sound pressure levels, in 2- and 9-month-old C57 mice. **B** ABR thresholds were measured from 2- and 9-month-old C57 mice at different frequencies (4–32 kHz). Arrows indicate the threshold exceeding the maximum of TDT ABR system. **C** Representative ABR waveforms of 2- and 9-month-old BALB/c mice. **D** ABR thresholds of 2- and 9-month-old BALB/c mice are indicated. **E** Immunofluorescence showed the morphological changes of HCs in 2- and 9-month-old C57 and BALB/c mice. Scale bar, 10 µm. **F** Quantification of OHCs and IHCs from 2- and 9-month-old C57 mice. **G** DPOAE threshold measurement of 2- and 9-month-old C57 mice. Arrows indicate the threshold exceeding the maximum of TDT system. **H** Quantification of OHCs and IHCs from 2- and 9-month-old BALB/c mice. **I** Morphological changes of SGNs were observed in the apical, middle, and basal cochlea of 2- and 9-month-old BALB/c and C57 mice. Scale bar, 20 µm. **J** The density of SGNs was quantified in 2- and 9-month-old C57 mice and BALB/c mice. Data are represented as means ± SEM, **P < 0.01, ***P < 0.001.

We first examined auditory function, via ABR, of 2- and 9-month-old C57BL/6 mice, hereafter referred to as C57, and BALB/c mice. Compared with 2-month-old C57 mice, 9-month-old C57 mice displayed significantly elevated ABR thresholds across all frequencies (Fig. 1A, B), indicating hearing decline. There was no statistical difference in hearing function between 2- and 9-month-old BALB/c mice (Fig. 1C, D). Given that C57 mice showed deafness at 9 months of age, we used 9-month-old C57 mice as the AHL mouse model.

Next, we examined the morphology of HCs and SGNs in 2- and 9-month-old C57 as well as BALB/c mice. As shown in Fig. 1E, a disorderly arrangement of HCs was observed in 9-month-old C57 mice. The number of inner HCs (IHCs) and outer HCs (OHCs) was reduced in apex, middle, and base of the cochlea (Fig. 1F). Further, we also measured the distortion product otoacoustic emissions (DPOAE) from 2-and 9-month old C57 mice. We demonstrated that DPOAE thresholds were significantly increased in 9-month-old mice (Fig. 1G), indicating a decline in the function of OHCs. In addition, 9-month-old C57 mice also showed a decrease in SGN density (Fig. 1I, J). However, we did not find any apparent morphological abnormality or loss of SGNs and HCs in the apical, middle, and basal turns of the cochlea in 2-month-old and 9-month-old BALB/c mice (Fig. 1E, H, I, J). These data indicate that the degeneration of SGNs and HCs might be responsible for hearing loss in 9-month-old C57 mice.

### Downregulation of REST in SGNs and HCs in AHL mice is associated with apoptosis

**Fig. 2:**
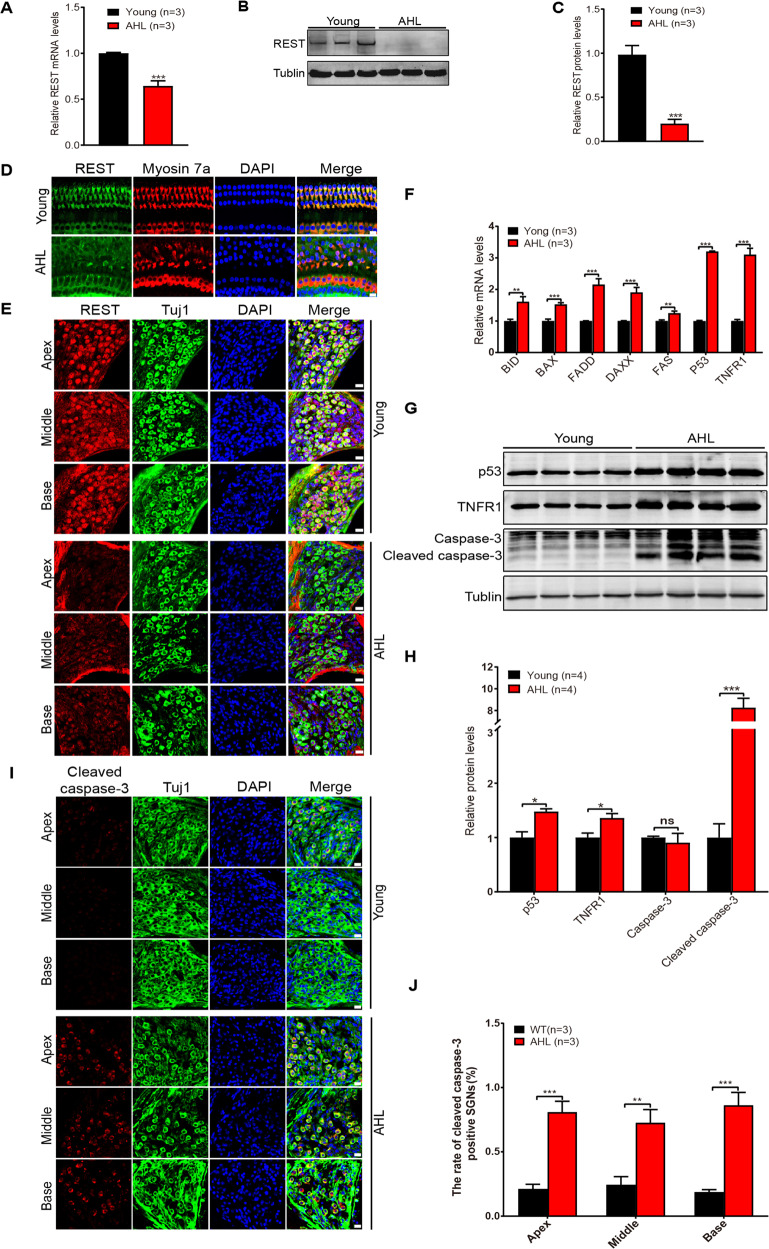
Downregulation of REST in SGNs and HCs in AHL mice is associated with apoptosis. **A**–**C** REST expression was significantly decreased in the cochleae of AHL mice at the mRNA and protein levels, as determined by real-time PCR (**A**) and Western blotting (**B**, **C**). **D**, **E** Immunofluorescence showed REST expression in HCs (**D**) and SGNs (**E**) from young and AHL mice. Scale bar: 10 µm in (**D**), 20 µm in (**E**). **F** The mRNA expression levels of BID, BAX, FADD, FAS, P53, TNFR1 were significantly increased in cochleae from AHL mice. **G**, **H** The protein levels of p53, TNFR1, caspase-3 and cleaved caspase-3 were increased in AHL mice, as determined by Western blot (*n* = 4). **I**, **J** Expression of cleaved caspase-3 in SGNs from young and AHL mice. Data are represented as mean ± SEM. **P < 0.01, ***P < 0.001.

To explore the role of REST in AHL, we examined the expression of REST in the cochlea of young (2-month-old) and AHL (9-month-old) C57 mice. The expression of REST at both the mRNA and protein level was significantly decreased in the cochlea of AHL mice (Fig. 2A–C). Immunofluorescence staining images showed the expression of REST on Myosin 7a^+^ cell was decreased, suggesting REST was downregulated in the HCs of AHL mice (Fig. 2D). Reduced expression of REST was also observed in SGNs at the apex, middle, and base of the cochlea, in AHL mice (Fig. 2E). Next, we examined the expression of cell death-related genes in the cochlea of young and AHL mice. As shown in Fig. 2F, the expression of p53, TNFR1, FAS, BID (BH3 interacting domain), BAX (Bcl2-associated X protein), FADD (Fas-associated death domain) and DAXX (death domain associated protein), at the mRNA level was significantly increased in AHL mice. The increased expression of p53 and TNFR1 in protein level was confirmed by western blotting (Fig. 2G, H). In addition, cleaved caspase-3 was detected in the cochlea tissue and SGNs of AHL mice (Fig. 2G–I). Further quantification showed the percentage of cleaved caspase-3 positive cells in AHL mice was significantly higher than in young mice (Fig. 2J). Taken together, these data indicated that REST was downregulated in AHL mice, which is associated with cell apoptosis in SGNs and HCs.

### Deletion of REST in cochlear results in hearing impairment in mice

**Fig. 3:**
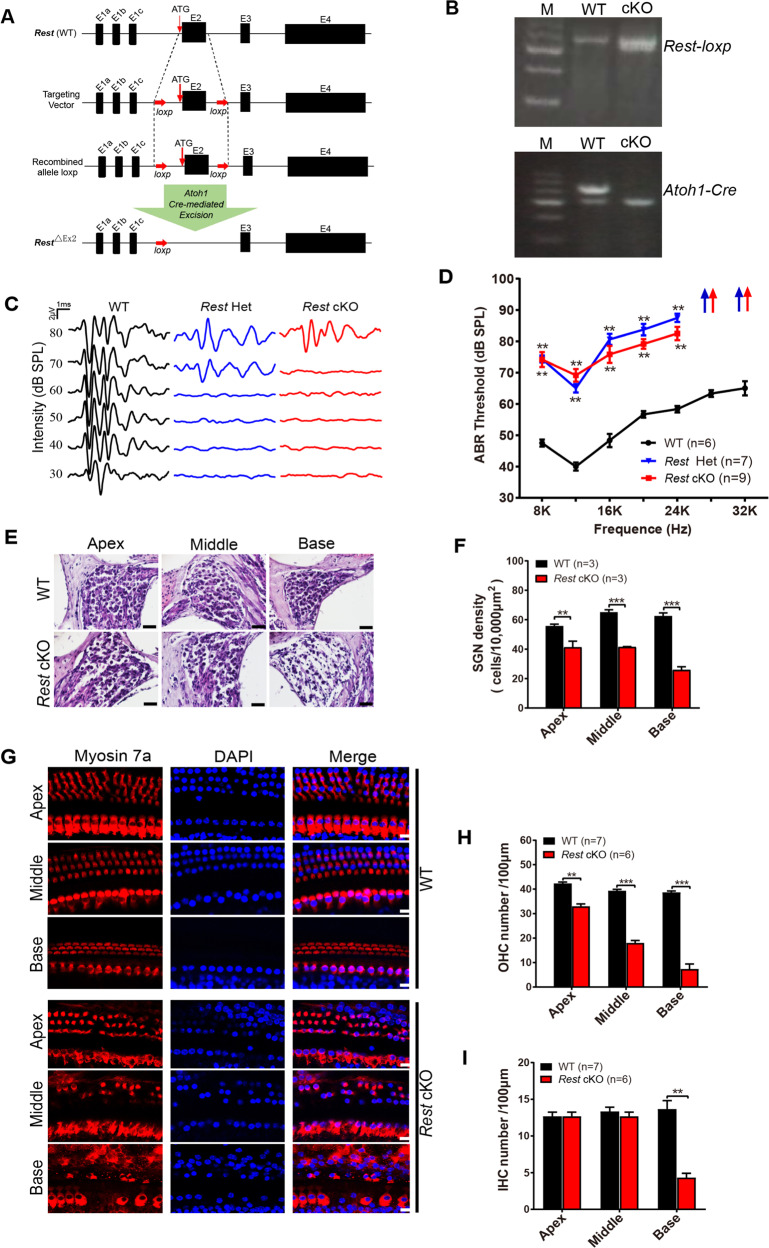
Deletion of REST in cochlear results in hearing impairment in mice. **A** Schematic illustration of the Rest conditional knockout (*Rest* cKO) generation process. Two LoxP sites are inserted on either side of the coding sequence of exon 2. Expression of Cre recombinase is activated by a specific *Atoh1* promoter in the cochlea, resulting in loss of REST function in the cochlea. **B** Modified *Rest* flox allele exon 2 and *Atoh1*-*Cre* insertion was confirmed by PCR genotyping of DNA extracted from cochlea of vehicle (WT), or *Rest* cKO mice. **C** Representative ABR waveforms of WT, *Rest* Het (Heterozygote mice), and *Rest* cKO (Homozygous mice). **D** ABR thresholds of WT, *Rest* Het, and *Rest* cKO mice in response to pure tone stimuli (8–32 kHz). **E** Morphological changes of SGNs from WT and *Rest* cKO mice. Scale bar: 20 µm. **F** The density of SGNs was quantified in WT and *Rest* cKO mice. **G** Morphological changes in HCs were observed from WT and *Rest* cKO mice. Scale bar: 10 µm. **H**, **I** Quantification of OHCs and IHCs in WT and *Rest* cKO mice. Data are represented as mean ± SEM. **P < 0.01, ***P < 0.001.

To investigate whether downregulation of REST causes hearing impairment, we performed conditional null deletion of *Rest* in SGNs and HCs. Mice with homozygous intronic LoxP sites, flanking exon 2 of *Rest* (*Rest*^*flox/flox*^) [23], were crossed with mice expressing the Cre recombinase transgene, under the *Atoh1*-specific promoter, to create *Rest* cKO mice (Fig. 3A). The genotypes of the pups were identified using PCR analysis (Fig. 3B). Compared to 3-month-old wild type (WT) mice, the hearing threshold was significantly higher in both age-matched heterozygous (*Rest* Het), and homozygous *Rest* cKO mice, they also exceeded the upper limit of ABR at high-frequency (28 and 32 kHz), suggesting that auditory function had declined in REST deficient mice (Fig. 3 C, D). Therefore, we used *Rest* homozygous mice in subsequent experiments.

We the further examined whether REST deficiency can damage SGNs and HCs in mice. As shown in Fig. 3E, F, all three regions of the cochlea, i.e., the apex, middle, and base, in *Rest* cKO mice, displayed severe loss of SGNs. Moreover, the number of OHCs and IHCs in the basal cochlea was also reduced in *Rest* cKO mice (Fig. 3 G–I), suggesting that deletion of REST causes degeneration of SGNs and HCs.

### REST deficiency induced apoptosis of SGNs and HCs in 3-month-old mice

**Fig. 4:**
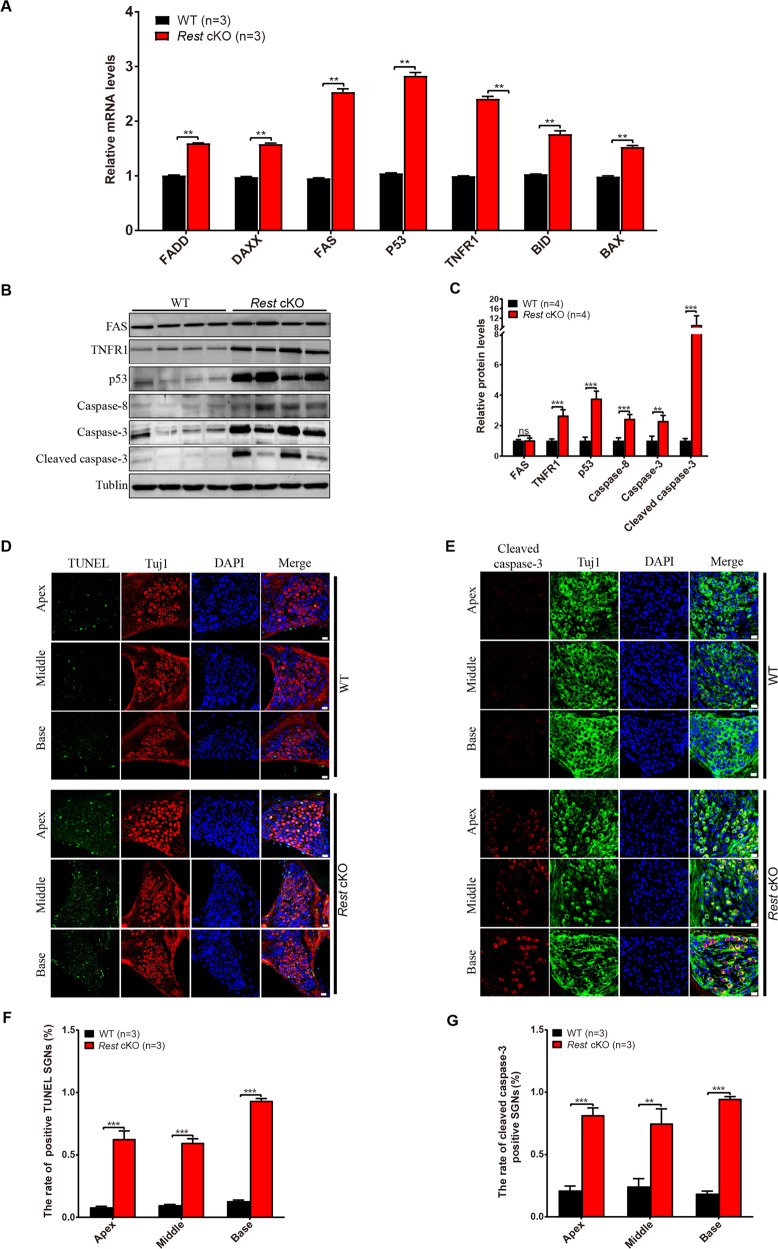
REST deficiency induced apoptosis of SGNs and HCs in 3-month-old mice. **A** mRNA levels of apoptosis-related genes showed an increase in the cochlea of *Rest* cKO mice. **B**, **C** Expression of apoptosis-related protein in cochlea from WT and *Rest* cKO mice was measured by Western blot. **D** TUNEL staining showed the apoptosis of SGNs in apex, middle, and base of the cochlea from WT and *Rest* cKO mice. Scale bar: 20 µm. **E** Expression of cleaved caspase-3 in cochlea from WT and *Rest* cKO mice. Scale bar: 20 µm. **F**, **G** Quantification of the number of TUNEL-positive cells and cleaved-caspase-3 cells in SGNs of WT and *Rest* cKO mice. Data are represented as mean ± SEM. **P < 0.01, ***P < 0.001.

We then determined whether REST deficiency induced loss of SGNs and HCs by apoptosis. Similar to AHL mice, mRNA levels of FADD, DAXX, FAS, p53, TNFR1, BID, BAX were increased in the cochlea of *Rest* cKO mice (Fig. 4A). Notably, P53, TNFR1 and FAS protein levels were dramatically elevated in *Rest* cKO mice (Fig. 4B). Subsequently, we examined the expression of apoptosis-related proteins in *Rest* cKO mice. As shown in Fig. 4B, C, an increase in caspase-8, caspase-3 and cleaved caspase-3 concentration was observed in the cochleae of *Rest* cKO mice. This was confirmed by the TUNEL assay and immunostaining in the SGNs of *Rest* cKO mice (Fig. 4D–G). Collectively, these data demonstrate that REST deficiency induces loss of SGNs and HCs by apoptosis.

### p53 inhibitor pifithrin-α rescues the hearing loss in *Rest* cKO mice

**Fig. 5:**
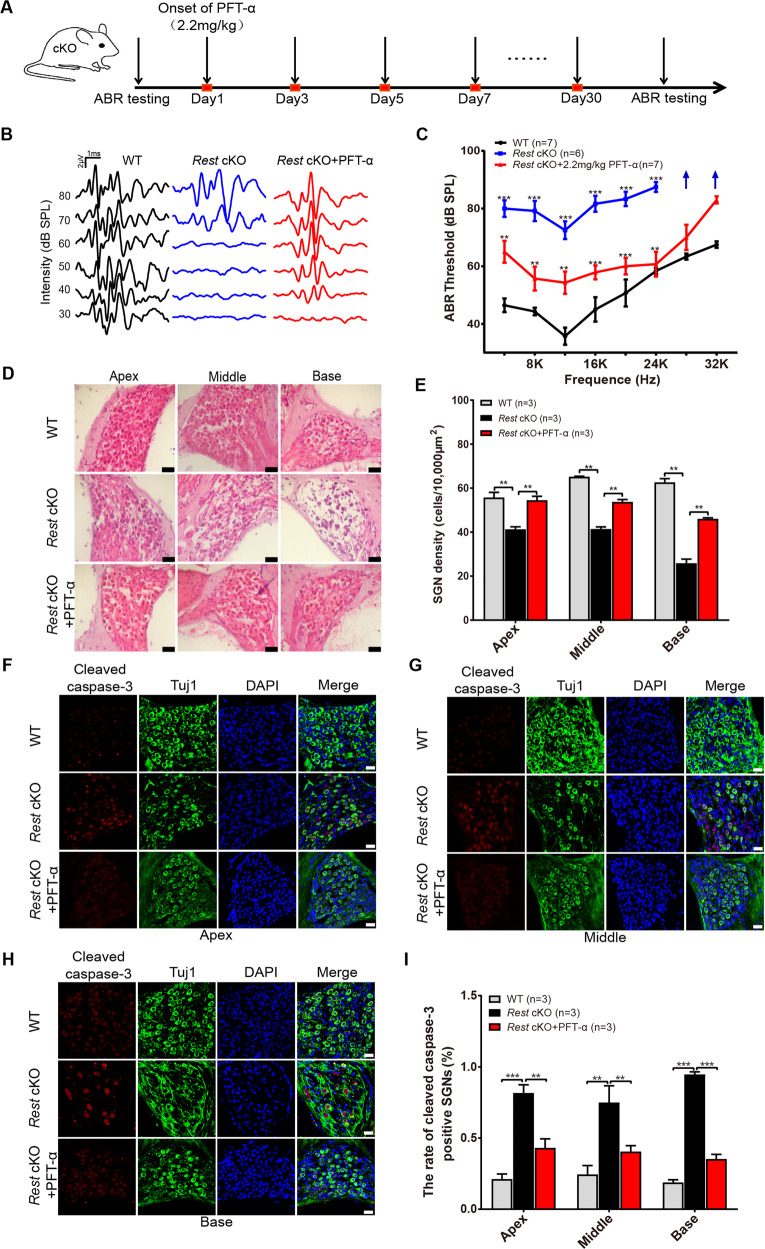
p53 inhibitor pifithrin-α rescues the hearing loss in *Rest* cKO mice. **A** Timeline for ABR testing and drug treatment. Pifithrin-α was administered to 2-month-old *Rest* cKO mice at 2.2 mg/kg every other day, for 30 days. **B** Representative ABR waveforms were shown in WT, *Rest* cKO, and *Rest* cKO+ pifithrin-α (PFT-α) mice. **C** ABR thresholds of WT and *Rest* cKO mice are treated with pifithrin-α. Arrows indicate when the threshold exceeded the upper limits of TDT ABR system. **D**, **E** Morphometry of SGNs in WT, *Rest* cKO, and *Rest* cKO+ pifithrin-α mice. Scale bar: 20 µm. **F**–**I** Expression of cleaved caspase-3 in SGNs of apex, middle and base of cochlea from WT and *Rest* cKO mice. Scale bar: 20 µm. Data are represented as mean ± SEM. **P < 0.01, ***P < 0.001.

Since P53 expression levels were most significantly increased in the cochlea of *Rest* cKO mice, we next examined whether the P53-mediated apoptotic pathway played a role in their deafness. We administered the p53 inhibitor pifithrin-α (i.p., 2.2 mg/kg every other day) to 2-month-old *Rest* cKO mice, for 30 days (Fig. 5A). Compared to a control group, the hearing threshold of *Rest* cKO mice treated with pifithrin-α was profoundly reduced, indicating an improvement in their hearing (Fig. 5B, C). We then determined the effect of pifithrin-α on apoptosis. We found that pifithrin-α significantly reduced the loss of SGNs in the *Rest* cKO mice (Fig. 5D, E). Additionally, the percentage of cleaved caspase-3-positive cells was decreased in the SGNs of *Rest* cKO mice treated with pifithrin-α, compared to control mice (Fig. 5F–I). These results suggest that REST deficiency may induce apoptosis in the cochlea via P53 and further contribute to hearing loss in mice.

### Upregulation of REST attenuates apoptosis by inhibiting the P53 pathway in vitro

**Fig. 6:**
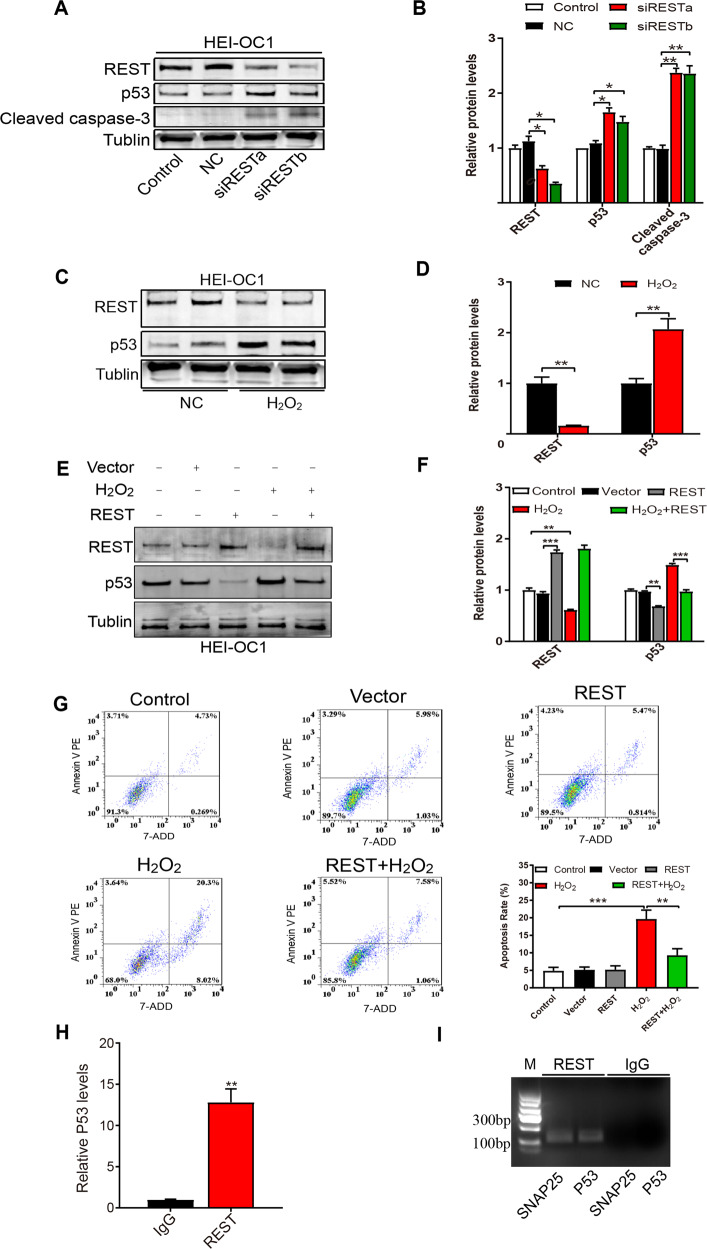
Upregulation of REST attenuates apoptosis by inhibiting the P53 pathway in vitro. **A**, **B** REST knockdown by siRNA increased p53 and cleaved caspase-3 expression in HEI-OC1 cells. **C**, **D**. The expression of REST and p53 in HEI-OC1 cells treated with H_2_O_2_ for 1 hour. **E**, **F** Expression of REST and p53 were measured in HEI-OC1 cells transfected with REST plasmid for for 24 h and/or treated with H_2_O_2_ for 1 hour. **G** Apoptosis of HEI-OC1 cells transfected with REST plasmid for 24 h and/or treated with H_2_O_2_ for 1 hour was determined by Flow cytometry. **H** ChIP analysis indicated the direct binding of REST to the promoter regions of p53 gene in HEI-OC1 cells. **I** ChIP assay detecting P53 and SNAP25 confirmed REST binding to p53 by nucleic acid electrophoresis, using isotype IgG as negative control. Data are represented as mean ± SEM. **P < 0.01, ***P < 0.001.

To verify the role of REST on regulating apoptosis via p53, we observed the effect of REST on P53 in vitro. Knockdown of REST by siRNA upregulated the expression of p53 and cleaved caspase-3 in an inner ear cell line, HEI-OC1 cells, in vitro (Fig. 6A, B).

Next, we treated the HEI-OC1 cells with H_2_O_2_ to construct an in vitro model of AHL. CCK-8 was used to determine the optimal concentration of H_2_O_2_ that resulted in a decrease in HEI-OC1 activity (Supplementary Figure 1). As shown in Fig. 6C, D, REST expression was reduced in the H_2_O_2_-induced in vitro model of AHL, with an increase in P53 expression. Furthermore, overexpression of REST inhibited the high expression of p53 protein and reduced apoptosis in H_2_O_2_-induced HEI-OC1 cells (Fig. 6E–G). We further investigated whether REST binds to its consensus site RE1 element on the p53 promoter. The ChIP assays revealed that REST could bind directly to its site on the p53 promoter (Fig. 6H, I). Thus, these results further confirm that REST deficiency induced apoptosis through upregulation of P53 in vitro.

### p53 inhibitor pifithrin-α rescues the hearing loss in AHL mice

**Fig. 7:**
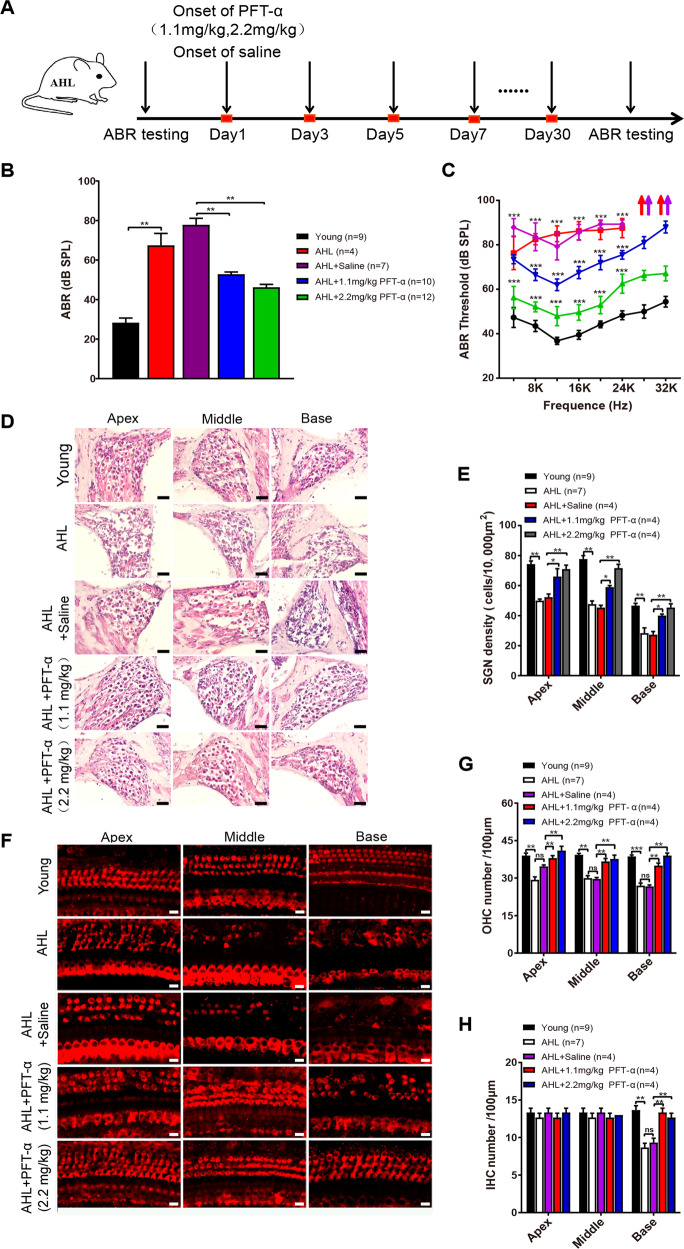
p53 inhibitor pifithrin-α rescues the hearing loss in AHL mice. **A** Timeline for ABR testing and drug treatment. Pifithrin-α was administered to 8-month-old WT mice at 1.1 or 2.2 mg/kg every other day, for 30 days. **B**, **C** ABR thresholds in response to click (**B**) or pure-tone stimuli (**C**) were applied in Young, AHL, AHL + saline, AHL + 1.1 mg/kg pifithrin-α (PFT-α), and AHL + 2.2 mg/kg (PFT-α) mice. **D**, **E** Changes in SGN morphology and density in Young, AHL, AHL + saline, AHL + 1.1 mg/kg PFT-α, and AHL + 2.2 mg/kg PFT-α mice. **F** Immunofluorescence images showed alterations of HCs in mice in different treatment groups. **G**, **H** Quantification of OHCs (**G**) and IHCs (**H**) at the cochlea’s apical middle and basal regions in Young, AHL, and PFT-α treated mice. Scale bar: 20 µm in D, 10 µm in (**F**). Data are represented as mean ± SEM. **P < 0.01, ***P < 0.001.

**Fig. 8:**
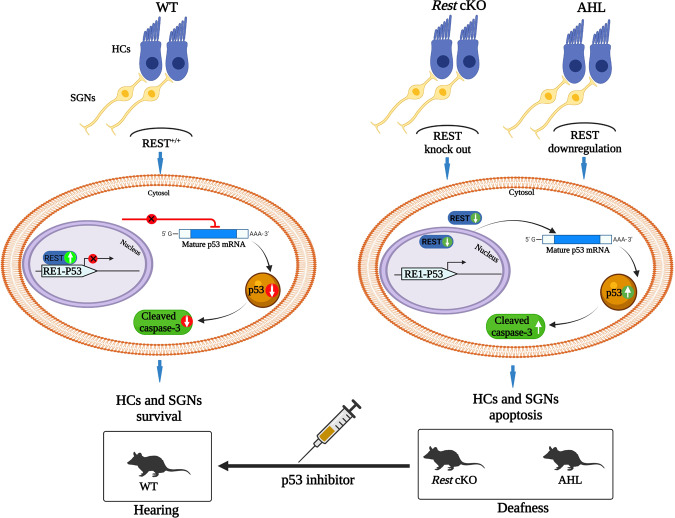
Model for mechanisms of REST downregulated induced apoptosis of SGNs and HCs through p53 pathway contribute to AHL. In WT mice, REST inhibits P53 expression, which allows HCs and SGNs exert their normal functions, and thus preserve proper hearing. In contrast, in the cochleae of *Rest* cKO mice or AHL mice, REST expression was decreased, resulting in reduced inhibition of P53. The high level of p53 leads to apoptosis of SGNs and HCs, and therefore hearing loss in those mice. Administration of p53 inhibitor improved hearing in *Rest* cKO and AHL mice.

Since REST-deficiency-induced p53 upregulation may trigger apoptosis in HEI-OC1 cells in vitro, we further investigated whether inhibition of p53 by pifithrin-α may reduce cochlear cell damage and improve hearing and in AHL mice. Pifithrin-α (i.p., 1.1 and 2.2 mg/kg, every other day) was administered to the 8-month-old C57 mice, for 30 days (Fig. 7A). Both low dose (1.1 mg/kg) and high dose (2.2 mg/kg) pifithrin-α significantly reduced the ABR threshold in AHL mice, compared to saline-treated mice (Fig. 7B, C), indicating a partial improvement in their hearing. In addition, pifithrin-α reduced SGN (Fig. 7D, E) and HC (Fig. 7F–H) damage, preserving their cell number―which is reduced in AHL (Fig. 8).

In summary, our findings suggest that decreased REST expression causes an upregulation of P53, leading to SGNs and HCs apoptosis, which can result in hearing loss, in old mice. Inhibition of p53 reduced damage to SGNs and HCs, and improved hearing in AHL mice.

## Discussion

REST, a transcriptional repressor, was initially described as a regulator of neural differentiation and development. Recent studies have revealed that nearly 2,000 genes contain predicted REST binding sites, and the protein controls many cellular processes fundamental to normal physiology, as well as pathological conditions [10, 24, 25]. Emerging evidence indicates that REST is an important player in age-related disease [26]. However, the role of REST in AHL is still unknown. Here, we found that the expression of REST was decreased in SGNs and HCs in AHL mice. The specific deletion of REST in the cochlea induced P53-dependent apoptosis of SGNs and HCs, and hearing decline, in mice. Meanwhile, p53 inhibitor pifithrin-α reduced the degeneration of SGNs and HCs and rescued the hearing deficit in AHL mice, suggesting REST is an important contributor to AHL.

The mammalian *Rest* gene consists of four exons and three introns. Nakano et al. reported that the deletion of exon 4 resulted in the gain-of-function of REST, which is associated with DFNA27 hearing loss [27]. Here, we used *Rest* exon 2-flox mice crossed with *Atoh1-Cre* mice to determine the effect of REST on hearing in mice. Exon 2 is an essential exon encoding a functional REST protein, the knockout of exon-2 results in loss of function of REST. The *Atoh1* gene is mainly expressed in SGNs and HCs in cochlea [28, 29], therefore, the *Atoh1-Cre* mice can be used as a tool to efficiently knockout *Rest* in SGNs and HCs. C57 mice show a typical pattern of AHL at 9-12 months of age, when mice are in middle age [30]. Deafness in C57 mice due to mutations in the *cdh23* gene, which encodes a component of the mechano-transducer apparatus, has been identified as an important contributor to AHL [31, 32]. Therefore, C57 mice can be used as an animal model for the early onset of age-related deafness. Our findings showed that loss of function of REST in SGNs and OHCs leads to hearing loss, indicating that a certain range of REST expression levels is necessary for normal hearing, and that reductions or increases outside of this “safe” range causes cochlear dysfunction.

Apoptosis of cochlear cells is a contributing factor in AHL [8, 33, 34]. Changes in REST expression were associated with apoptosis. High levels of REST in cortical neurons of aged individuals inhibited neuron excitability, activated the longevity genes FOX1 and DAF16, and attenuated neuron degeneration [18]. Reduced REST expression induced a loss of prefrontal cortical neurons in patients with AD through upregulation of multiple genes including FAS, FADD, TRADD, BAX, DAXX, and PUMA [16]. Here, we found that, unlike genes involved in cortical neuronal apoptosis [16], p53 was significantly upregulated in cochlea of AHL mice, suggesting that REST induced apoptosis through the upregulation of p53. This was further confirmed by the elevated p53 expression seen in the cochlea of *Rest* cKO mice. The endogenous p53 pathway, induced by stresses such as acute DNA damage, plays a critical role in apoptosis, cell growth and senescence [35–37]. p53 induces cell apoptosis through different methods, inducing the self-cleavage and activation of downstream caspase-3 [38]. In addition, p53-mediated cell apoptosis has been determined by p53 acetylation on Lys-120 residue [39].

To verify that downregulated REST induces apoptosis through the p53 pathway, we built an aging cell model with H_2_O_2_ and further investigated the role of REST in regulating p53 during aging in vitro. Regarding the molecular basis of aging, oxidative stress is thought to be the most redundant trigger of cellular aging and senescence [40]. H_2_O_2_ is the most common agent inducing senescence because it naturally induces oxidative stress, which can cause premature senescence in auditory cells, resulting in AHL [41]. The model of senescence induced by H_2_O_2_ in the HEI-OC1 cell lines, can be a useful tool to study the intrinsic mechanisms responsible for AHL in vitro [42]. In this study, we found that REST knockdown by siRNA increases the expression of p53 and cleaved caspase-3 in HEI-OC1 cells. In H_2_O_2_-induced aging HEI-OC1 cells, REST was downregulated and associated with p53 upregulation. Furthermore, the overexpression of REST could decrease p53 expression and protect against cell apoptosis in the aging HEI-OC1 cells. Intriguingly, we found that REST can bind to the predicted RE-1 binding site of the p53 promoter, further supporting the idea that REST deficiency can upregulate p53 to trigger apoptosis.

Considering that p53 is a key molecule mediating H_2_O_2_-induced senescence in HEI-OC1 cells [40, 43], REST defects may be dependent on p53-mediated apoptotic pathways that lead to AHL. Our results demonstrate that administration of p53 inhibitor pifithrin-α attenuated impairment of SGNs and HCs in cochlea, and improved hearing in AHL mice. The pifithrin-α alleviated cardiac aging and enhanced fatty acid metabolism in the aged *Rap1*^*−/−*^ mice, suggesting p53 signaling could represent a therapeutic target in preventing/attenuating cardiac aging [44]. Therefore, we predict that the p53 also has the potential to be an intervention target for AHL.

In conclusion, our findings revealed that a certain level of REST expression is required for proper auditory functions in aging mice. REST deficit in the cochlea of aged mice can induce apoptosis of SGNs and HCs through the upregulation of p53, which in turn results in AHL. Our study contributes to understanding of the essential functions of REST in hearing during aging. It provides new insight for potential future treatment of AHL.

## Supplementary information

supplemental figure legend

supplemental figure 1

uncropped western blot images

reproducibility checklist

## Data Availability

All data generated in the study are included in this article.
