# Supplementary material for: Downregulation of REST in the cochlea contributes to age-related hearing loss via the p53 apoptosis pathway
Source: Cell Death Dis. 2022 Apr 13;13(4):343. doi: 10.1038/s41419-022-04774-0 (PMC9007975; doi:10.1038/s41419-022-04774-0)
Supplement: Supplementary file 1 — supplemental figure legend [file 41419_2022_4774_MOESM1_ESM.doc]

**Suppl Figure.1 Effect of H_2_O_2_ on viability of HEI-OC1 cell.** HEI-OC1 cells were treated with H_2_O_2_ and cell viability was examined by CCK-8 assay at different time point. Data are mean ± SEM. **P<0.01, ***P<0.001.
