# Supplementary figures and images for: Downregulation of REST in the cochlea contributes to age-related hearing loss via the p53 apoptosis pathway
Source: Cell Death Dis. 2022 Apr 13;13(4):343. doi: 10.1038/s41419-022-04774-0 (PMC9007975; doi:10.1038/s41419-022-04774-0)

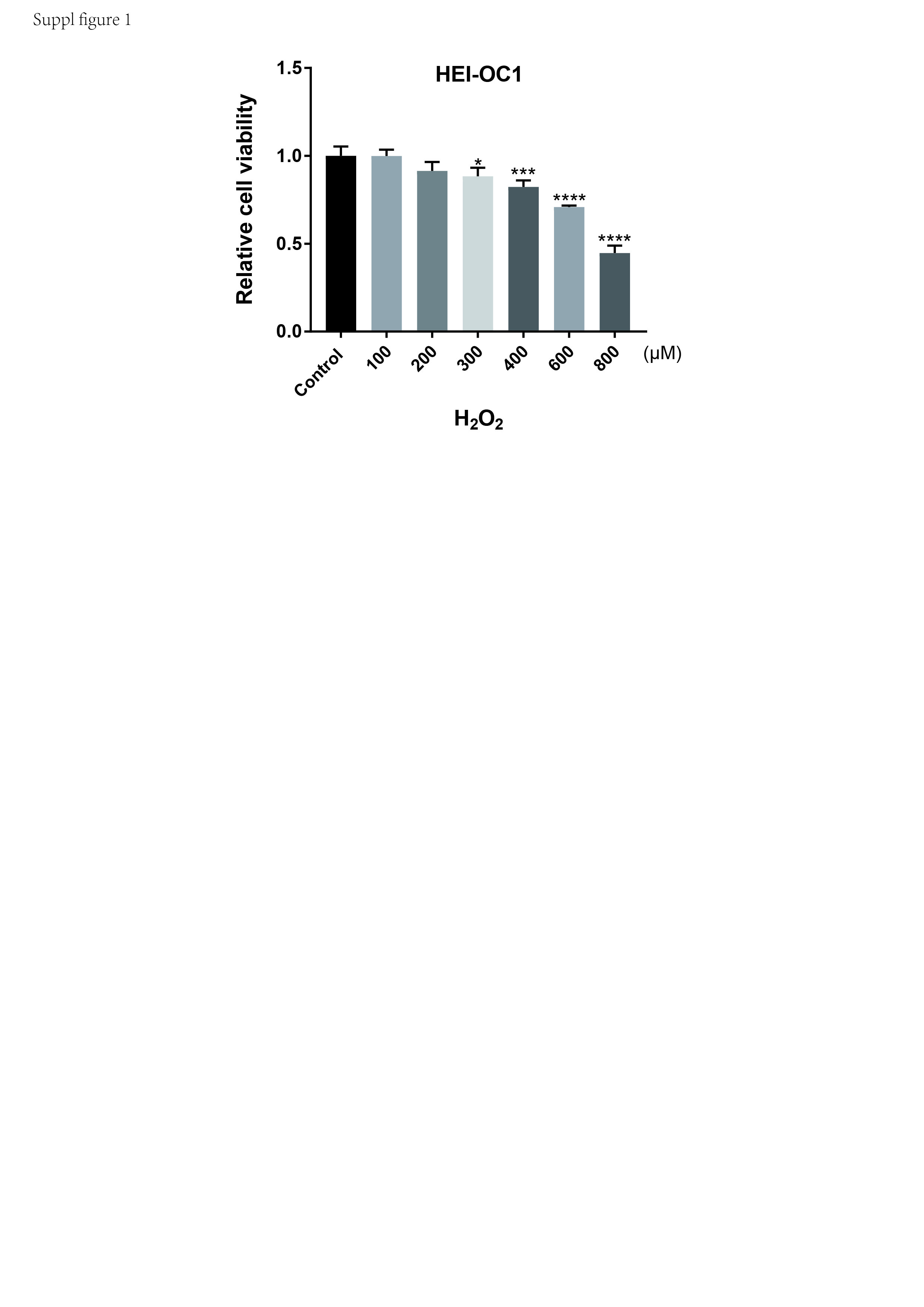

Supplement: Supplementary file 2 — supplemental figure 1 [file 41419_2022_4774_MOESM2_ESM.jpg]

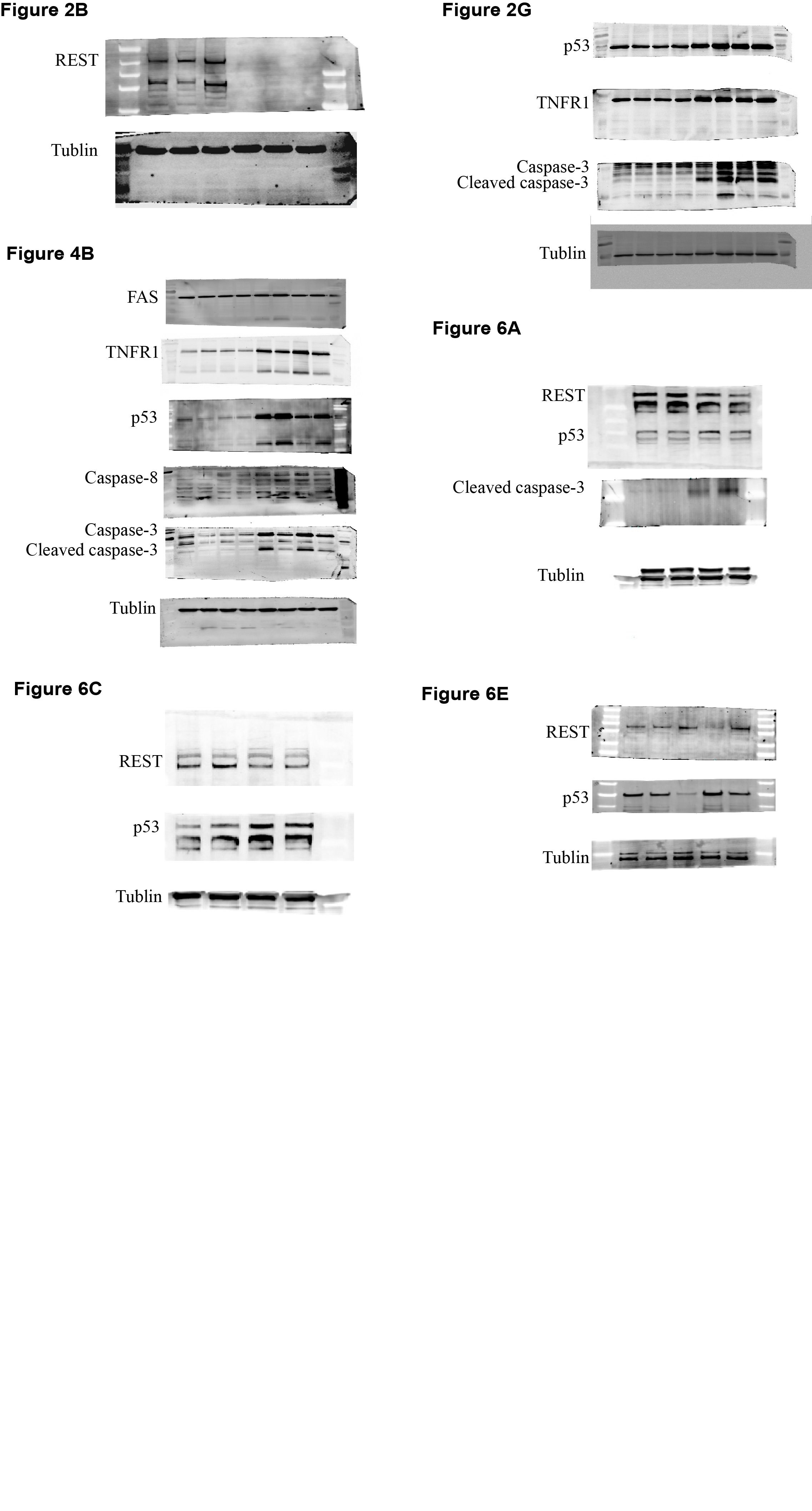

Supplement: Supplementary file 3 — uncropped western blot images [file 41419_2022_4774_MOESM3_ESM.jpg]
